# Supplementary material for: Vitamin D status, nutrition and growth in HIV-infected mothers and HIV-exposed infants and children in Botswana
Source: PLoS One. 2020 Aug 13;15(8):e0236510. doi: 10.1371/journal.pone.0236510 (PMC7425960; doi:10.1371/journal.pone.0236510)
Supplement: Attachment — Submitted filename: PONE-D-20-05581.pdf [file pone.0236510.s002.pdf]

PONE-D-20-05581

Vitamin D status, nutrition and growth in HIV-infected mothers and HIV-exposed infants and children in Botswana

Alyssa M Tindall *et al.*

The authors present a cross-sectional evaluation of vitamin D status in 36 (?) women living with HIV and their HIV -exposed and -infected infants and children.

The methodology and results as presented are unclear and the manuscript does not meet the standards for publication in its current format.

Methods:

Much more detail is required to assess the study. For example:

1. How was the sample size of 36 determined?

Are all the infants and children drawn from these 36 women or is the sample actually 84? Again, how was this determined?

2. How were these women recruited? Were they attending routine adult HIV services and the asked about off-spring? Were the infants/children recruited first? Was the sample selected randomly? You balanced children >12m living with and without HIV. How was this done practically, and could it not introduce bias? If the women/children were recruited in a different manner, this could bias the results (we know already they were recruited in different seasons).

Is the hospital site a reflection of the population as a whole? You note that it is an urban population. This limitation should be discussed. As should the omission of all acutely or chronically unwell children who may have a different vitamin D status. It should be clearly stated that these findings are limited to quite a specific group.

3. With all the stratification the numbers become very small and once must be careful not to overstate the findings. Caution is required in interpreting these data.

4.The authors mention nutritional status and feeding in the introduction but seem to have little data on this. Only length/height is discussed and only breast feeding as a binary variable. Duration of breast feeding may also be relevant; age of introduction of other foods, variety of foods etc. It is better not to introduce these terms if you have no related data. No weight data are presented.

5. There are some more specific inconsistencies/lack of clarity in the methods section:

Line 120: HIV status – this is usually positive, negative or indeterminate; you may mean the clinical and/or immunological stage of women living with HIV?

Line 126: please specify which measurements

Lines 132 – 134; Both CDC and WHO systems are referenced. For clarity, please select one and use throughout.

Please add some information on the blood draws

Line 139: ‘Immunological outcomes’, like ‘an immune panel’ in abstract and ‘immunological markers’ in line 199 is a bit misleading if you mean CD4 and VL only. The terms imply additional assays (cytokines? T cell phenotype?)

Was blood taken for these assays at the same time as for the vit D assays? Or were these results collected from the records?

Line 154: were all data normally distributed?

Why did you choose ANCOVA over regression analysis? There are additional confounders (sex, breast-feeding, age at diagnosis, maternal ART, infant ART etc.) that could have an effect.

Did you check the model?

Please add a description of relevant PMTCT and ART guidelines at the time as these speak to additional exposures.

Results:

The presentation of the results in the text and the tables in unclear. The data also appears incomplete as associations are alluded to in the text for which there are no numbers. Please present the values and uncertainty ranges where appropriate.

Line 169: see confusion about the number of participants above.

Line 179: please clarify whether all/most women with infants living with HIV were seen in one season and all/most HEU infants and children in another?

Lines 181 – 184: is it possible to include all co-variables and present a final model as opposed to piece-meal? The authors peak to significance and adjustment for confounding but do not present the data from the model. It is possible to put some of these results with and without adjustment in a table or at least report them in the text?

Line 189: breastfeeding. Does this refer to current BF or ever BF?

Line 191: Please report these values and the uncertainty ranges to support thus statement.

Line 201: Are these CD4 values for the mothers or the infants/children? Conventionally, CD4 is presented as an absolute number in adults (and children >5years) and only as % in the under-fives.

Line 204: positively associated with higher or lower CD4 count?

Table 1: For continuous variables, please include *mean (SD)* somewhere so we know this is what is being presented.

For the categorical variables, can you include n (%)

The above applies to all tables

You mentioned EFV-based regimens as a potential confounder (line 184)– is the LSM also adjusted for this?

Discussion:

Given the concerns of the methodology and incompleteness of the results, it is difficult to assess the discussion. In general, I feel the results are over-stated given the small sample size and risks of bias. All limitations should be acknowledged and discussed. While the correlation co-efficients are significant the correlations are weak.

Line 238 etc. did all the studies use the same cut-off for vit-D deficiency?

Line 292: HIV disease status – only CD4 count is reported.
