# Supplementary material for: Vitamin D status, nutrition and growth in HIV-infected mothers and HIV-exposed infants and children in Botswana
Source: PLoS One. 2020 Aug 13;15(8):e0236510. doi: 10.1371/journal.pone.0236510 (PMC7425960; doi:10.1371/journal.pone.0236510)
Supplement: Attachment — Submitted filename: Bots-D Response to Reviewers 050620.docx [file pone.0236510.s003.docx]

PONE-D-20-05581
Vitamin D status, nutrition and growth in HIV-infected mothers and HIV-exposed infants and children in Botswana
PLOS ONE

Dear Dr. Tindall,

Thank you for submitting your manuscript to PLOS ONE. After careful consideration, we feel that it has merit but does not fully meet PLOS ONE’s publication criteria as it currently stands. Therefore, we invite you to submit a revised version of the manuscript that addresses the points raised during the review process.

==============================
Thank you for your submission. Please could you respond to the reviewer comments and to those in the attached pdf.

All reviewers have requested more detail of the methodology and results; the queries are listed in the reviews and pdf.

==============================

We would appreciate receiving your revised manuscript by May 14 2020 11:59PM. When you are ready to submit your revision, log on to <https://www.editorialmanager.com/pone/> and select the 'Submissions Needing Revision' folder to locate your manuscript file.

If you would like to make changes to your financial disclosure, please include your updated statement in your cover letter.

To enhance the reproducibility of your results, we recommend that if applicable you deposit your laboratory protocols in protocols.io, where a protocol can be assigned its own identifier (DOI) such that it can be cited independently in the future. For instructions see: <http://journals.plos.org/plosone/s/submission-guidelines#loc-laboratory-protocols>

Please include the following items when submitting your revised manuscript:

- A rebuttal letter that responds to each point raised by the academic editor and reviewer(s). This letter should be uploaded as separate file and labeled 'Response to Reviewers'.
- A marked-up copy of your manuscript that highlights changes made to the original version. This file should be uploaded as separate file and labeled 'Revised Manuscript with Track Changes'.
- An unmarked version of your revised paper without tracked changes. This file should be uploaded as separate file and labeled 'Manuscript'.

Please note while forming your response, if your article is accepted, you may have the opportunity to make the peer review history publicly available. The record will include editor decision letters (with reviews) and your responses to reviewer comments. If eligible, we will contact you to opt in or out.

We look forward to receiving your revised manuscript.

Kind regards,

Emma K. Kalk
Academic Editor
PLOS ONE

Journal Requirements:

When submitting your revision, we need you to address these additional requirements:

1. Please ensure that your manuscript meets PLOS ONE's style requirements, including those for file naming. The PLOS ONE style templates can be found at <http://www.plosone.org/attachments/PLOSOne_formatting_sample_main_body.pdf> and <http://www.plosone.org/attachments/PLOSOne_formatting_sample_title_authors_affiliations.pdf>

2. PLOS requires an ORCID iD for the corresponding author in Editorial Manager on papers submitted after December 6th, 2016. Please ensure that you have an ORCID iD and that it is validated in Editorial Manager. To do this, go to ‘Update my Information’ (in the upper left-hand corner of the main menu), and click on the Fetch/Validate link next to the ORCID field. This will take you to the ORCID site and allow you to create a new iD or authenticate a pre-existing iD in Editorial Manager. Please see the following video for instructions on linking an ORCID iD to your Editorial Manager account: <https://www.youtube.com/watch?v=_xcclfuvtxQ>

Reviewers' comments:

Reviewer's Responses to Questions

**Comments to the Author**

1. Is the manuscript technically sound, and do the data support the conclusions?

The manuscript must describe a technically sound piece of scientific research with data that supports the conclusions. Experiments must have been conducted rigorously, with appropriate controls, replication, and sample sizes. The conclusions must be drawn appropriately based on the data presented. 

Reviewer #1: Yes

Reviewer #2: No

2. Has the statistical analysis been performed appropriately and rigorously? 

Reviewer #1: Yes

Reviewer #2: No

3. Have the authors made all data underlying the findings in their manuscript fully available?

The [PLOS Data policy](http://www.plosone.org/static/policies.action#sharing) requires authors to make all data underlying the findings described in their manuscript fully available without restriction, with rare exception (please refer to the Data Availability Statement in the manuscript PDF file). The data should be provided as part of the manuscript or its supporting information, or deposited to a public repository. For example, in addition to summary statistics, the data points behind means, medians and variance measures should be available. If there are restrictions on publicly sharing data—e.g. participant privacy or use of data from a third party—those must be specified.

Reviewer #1: Yes

Reviewer #2: No

4. Is the manuscript presented in an intelligible fashion and written in standard English?

PLOS ONE does not copyedit accepted manuscripts, so the language in submitted articles must be clear, correct, and unambiguous. Any typographical or grammatical errors should be corrected at revision, so please note any specific errors here.

Reviewer #1: Yes

Reviewer #2: No

5. Review Comments to the Author

Please use the space provided to explain your answers to the questions above. You may also include additional comments for the author, including concerns about dual publication, research ethics, or publication ethics. (Please upload your review as an attachment if it exceeds 20,000 characters)

**Reviewer #1:** The manuscript titled: “Vitamin D status, nutrition and growth in HIV-infected mothers and HIV-exposed infants and children in Botswana.” presented by Tindall et al is reviewed below.

The authors present a paper about vitamin D status and its relation to growth and HIV disease state in women living with HIV and exposed infants. It is a cross-sectional quantitative study. The authors demonstrate that vitamin D insufficiency was relatively rare but that the level of vitamin D did correlate with growth and HIV indicators in mothers.

The information is useful and the work seems to be scientifically sound. The paper is very well written and clear but there are a few issues to address

Methods:
Line 95: Please consider adding details about where mothers were recruited from, was it from their ART follow-up clinics?

**Thank you for this suggestion. We have changed the previous sentence to read: Mothers, infants and children were recruited from Princess Marina Hospital, Gaborone, and Bamalete Lutheran Hospital, Ramotswa, in Botswana where they were followed for medical care.**

Line 111-112: The sentence “the younger infants” seems to conflict with the results in that the sentence says that PCR results were not yet available yet the results clearly distinguish positive and negative. I would say that you cannot present the results as “neg” vs “pos” infants if the negative group actually is largely unknown in terms of HIV status. Please also explain why HIV testing was not offered as part of the study if results were not available on enrolment.

**The HIV PCR testing was provided by the well-established, Botswana Pediatric HIV Program and the study was designed and budgeted for utilizing the results provided through this program. The Botswana 2013 Global AIDS Response Report (when study data were collected) reported that 47% of infants born to HIV positive mothers received a virological HIV test within two months after birth, this is likely why at the time of data collection, the younger infants did not have available results. However, the report also documents that the rate of transmission from mother to child at this time was less than 3%. Given the low transmission rates in this population, these infants were categorized as HIV- at the time of data collection.**

**Further, when we removed the infants who did not have a confirmatory test result from the analysis, the significance of the results remained the same for both the analysis between mothers with HIV+ and HIV- infants and between HIV+ and HIV- infants. Specifically, we performed analysis of the data for a subsample of the older infants (6-11.9 months old, n=18) for whom HIV status was known (10 were HIV- and 8 were HIV+). The analyses for this subgroup confirmed our findings presented in the paper: 1) mothers of HIV+ infants had significantly lower 25(OH)D levels and significantly lower CD4% than mothers of HIV- infants; 2) HIV+ infants had poorer growth status than their HIV- counterparts by almost 1 Z score for both length and weight Z scores, although likely due to small sample size this did not reach statistical significance.**

Line 114: The 48 children, were they at all related to the infants and mothers? Were they older siblings or totally unrelated. Please clarify also how and where they were recruited.

**The children enrolled in this study were unrelated to the mothers and infants. We have clarified this in the methods section: This was an observational, age- and HIV-status stratified survey of 36 HIV-infected (HIV+) mothers and their infants (0-11.9 months), and 48 unrelated children (1-7.9 years).**

Line 132-133: In terms of using both the CDC as well as WHO charts, please explain why and also whether this may cause an effect on the results.

**This is the recommendation of the CDC – the CDC recommends using WHO growth charts for children 0-2 years and the CDC growth charts for children 2-18 years. We have amended the sentence in our methods to read: For infants and young children up to age two years where length was measured, length and weight were converted into Z scores (standard deviation units) using the WHO growth chart data (**[**http://www.cdc.gov/growthcharts/**](http://www.cdc.gov/growthcharts/)**), as recommended by the CDC(18). For older children the CDC recommends using the CDC algorithms to convert height and weight into Z scores. Given this is the recommendation for these age groups, we suggest would be inappropriate to use one of the reference charts for all ages.**

Discussion:
Line 248-249 and also line 264-265: Please consider explaining the relationship between cause and effect of Vit D and various clinical outcomes. Is there evidence that clearly links vitamin D as the cause and height as the effect?

**Thank you for this question. The relation between vitamin D and height is being explored in other clinical research settings. A recently published prospective study examining the effect of micronutrient status and growth in exclusively breastfed children reported:
“Children who were exclusively breastfed for longer than 4 months without proper supplement were more likely to have persistent vitamin D insufficiency and have relatively slower growth after infancy compared to mix-fed children.” 
Although these children were not exposed to HIV, there was still a relation between vitamin D status and height. The children in this study were Taiwanese and in the discussion of our study, we present studies that report on populations closer to the population we studied to compare vitamin D status and growth, particularly in HIV exposed infants and children.**

Line 273-274: What comparison are you using the make this statement “Our results also..”

**Thank you for this comment, we have changed the sentence to read: Overall, our results show HIV-exposed infants and children had poorer overall growth regardless of whether they were HIV- or HIV+.**

Minor:
Line 45: consider removing one “and” and replace with comma,

**We have changed the sentence to read: Length/height and weight Z scores determined growth status.**

Line 86: add the word “vitamin”

**We have added this.**

Line 262: the word “disease” seems to be missing after HIV.

**We have added this.**

Line 302: remove “the”

**We have removed this.**

**Reviewer #2:** The authors are presenting a research study which was designed to determine the vitamin D status in HIV-infected mothers and their children, and to compare vitamin serum levels between HIV positive and negative children.

Major comments
1. The number of participants enrolled is small, only 36 mothers and 48 children. In addition to these small numbers the children of the 36 HIV positive numbers are further divided to HIV-infected to HIV-exposed, with only 9 being HIV infected. This makes it difficult to interpret the significance of findings in this study. How was the sample size calculated, and what were the assumptions made to calculate the sample size?

**The primary objective of this study was to determine the vitamin D status of HIV-infected mothers and their infants and children. This was an observational study or survey of vitamin D in Botswana mothers, infants and children. Sample sizes were not calculated based upon effect sizes for the outcomes. Secondary objectives were to examine potential differences in vitamin D status and other outcomes among each of the groups and associations were exploratory.**

2. In children, they have stratified them to HIV-positive and HIV-negative, why was the same stratification not done for the mothers?

**Thank you for this question. All of the mothers were HIV+ by study design, so the same stratification is not possible.**

3. In presenting the Tables, authors have pointed on variables that showed statistical significance using different symbols. It will be much easier for readers to understand the Tables better if they can add a column with p-values.

**We have added the p-values to the table and removed the symbols that described the significance level.**

4. In Table 2, for the differences notes in length and height Z scores, did they adjust for potential confounders e.g. sex, and breast feeding.

**Thank you for this question. We did examine males and females separately and they had similar growth outcomes. We did adjust for age in the analysis of covariance as this was significant when added to the model. When added to the models, neither sex nor breastfeeding (ever breastfed) were significant covariates, that is, they did not significantly contribute to the growth outcomes and therefore we did not include them in the final model. It is worth noting that very few infants were breastfed (2 in the HIV- group and 1 in the HIV+ group). For the older children, there were no HIV- children who were ever breastfed. A little over half of the HIV+ children had been breastfed. Therefore, we examined using student’s unpaired t tests whether there were differences in growth (length and weight Z scores) among HIV+ children only who were breastfed vs not breastfed and found no significant differences by breastfeeding. Since breastfeeding only occurred in the HIV+ children, we cannot rule out that it may have contributed to the poorer growth status in these children along with other factors, and further studies are be needed to tease this out.**

5. Table 3 does not reflect on what was being studied, that is vitamin D. Secondly the numbers presented for HIV positive infants and children are confusing. For example for CD4 count there are 2 values, what does this mean. On the row of CD4% the numbers in brackets (8) and (24) are referring to what?

**Thank you for this comment. Table 3 was included to provide the information on HIV disease status (both immunological and clinical characteristics) for the whole sample. Mothers of HIV exposed (but HIV-) and HIV+ infants could be compared, and mothers of HIV+ infants had poor immunological status (lower CD4%). Therefore, we examined the association in these HIV-infected mothers of CD4% with vitamin D concentration, our primary outcome, and found a significant correlation.**

6. In making their conclusion, they need to adjust for potential confounders before they can conclude that children with HIV had poorer linear growth than HIV-uninfected children.

**We appreciate this comment and understand the reviewer’s concern. We have amended the language in the conclusion to address the fact that there may be confounding variables. Breastfeeding may be a confounding variable and a larger sample size and more even distribution amongst children who were breastfed vs formula fed in each group would lend to a better understanding. Additionally, we do not have dietary data to adjust for dietary intake and have listed this as a limitation of this study. We have also suggested future studies should examine confounding factors that could affect this relation as currently, we do not know what other variables may be confounders and this study is not designed for subgroup analyses.**

7. The conclusion that there was a significant association between vitamin D status and HIV disease is supported by which results?

**This conclusion is from the correlation between the HIV-infected mothers and CD4%: In the HIV-infected mothers as a group, serum 25(OH)D was positively associated with CD4% (r=0.40, p=0.016).**

8. The authors need to reconsider their statement that the findings from this study show a 'REASSURINGLY LOW PREVALENCE" of vitamin D insufficiency, when they had such a low sample size.

**We appreciate this suggestion. We have amended the sentence to: Results from this study show a low prevalence of vitamin D insufficiency in this small cohort and emphasize the need for further research to identify confounding variables that affect vitamin D status in HIV infected and exposed individuals.**

Minor comment
They must try and paraphase some of the sentences in the report as they are often long and one has to read them many times before one can understand their meaning. Below are some of the sentences to be considered for paraphrasing.
1. On page 4, Lines 80-83
2. On page 5, Lines 95-98
3. On page 9, Lines 180-184

**We have restructured these sentences to be shorter and read easier.**

6. PLOS authors have the option to publish the peer review history of their article ([what does this mean?](https://journals.plos.org/plosone/s/editorial-and-peer-review-process#loc-peer-review-history)). If published, this will include your full peer review and any attached files.


If you choose “no”, your identity will remain anonymous but your review may still be made public.


**Do you want your identity to be public for this peer review?** For information about this choice, including consent withdrawal, please see our [Privacy Policy](https://www.plos.org/privacy-policy).

Reviewer #1: No

Reviewer #2: No


[NOTE: If reviewer comments were submitted as an attachment file, they will be attached to this email and accessible via the submission site. Please log into your account, locate the manuscript record, and check for the action link "View Attachments". If this link does not appear, there are no attachment files to be viewed.]


While revising your submission, please upload your figure files to the Preflight Analysis and Conversion Engine (PACE) digital diagnostic tool,<https://pacev2.apexcovantage.com/>. PACE helps ensure that figures meet PLOS requirements. To use PACE, you must first register as a user. Registration is free. Then, login and navigate to the UPLOAD tab, where you will find detailed instructions on how to use the tool. If you encounter any issues or have any questions when using PACE, please email us at [figures@plos.org](mailto:figures@plos.org). Please note that Supporting Information files do not need this step.
