# Supplementary material for: Vitamin D status, nutrition and growth in HIV-infected mothers and HIV-exposed infants and children in Botswana
Source: PLoS One. 2020 Aug 13;15(8):e0236510. doi: 10.1371/journal.pone.0236510 (PMC7425960; doi:10.1371/journal.pone.0236510)
Supplement: Attachment — Submitted filename: Response to Editor 061120.docx [file pone.0236510.s004.docx]

Additional Editor Comments:

PONE-D-20-05581R1
Line 46: “Immune panel” implies more than CD4 count

**CD4, CD8 and viral load were also measured (HIV-1 RNA VL). We describe the methods in lines 137-145.**

Line 43: The groups of infant v child participants have been clarified in the text. Please do so in the abstract.

**We have clarified this: This was a cross-sectional study of HIV+ mothers and HIV-exposed infants and unrelated children (1-7.9 years).**

Line 80: Climate and sunlight are referenced; this could be applied to Botswana. There is already some data on vit D in pregnant women and children and HIV published by your group. you could mention this here.

**We have included an additional citation and sentence here: Vitamin D deficiency is more prevalent in children with perinatally acquired HIV infections(15).**

Line 89: The authors mention nutritional status and feeding in the introduction but seem to have little data on this. Only length/height is discussed and only breast feeding as a binary variable. Duration of breast feeding may also be relevant; age of introduction of other foods, variety of foods etc. It is better not to introduce these terms if you have no related data.

**We agree, there is a lot of nutritional information that would be helpful in assessing this population. We have amended the sentence to include “breastfeeding” instead of “nutrition”.**

Line 110: Sampling. Please provide more detail on how were these women recruited? Were they attending routine adult HIV services and the asked about off-spring? Were the infants/children recruited first? Was the sample selected randomly? Children >12m living with and without HIV were balanced. How was this done practically, and could it not introduce bias? If the women/children were recruited in a different manner, this could bias the results (we know already they were recruited in different seasons).
Is the hospital site a reflection of the population as a whole? You note that it is an urban population. This limitation should be discussed. As should the omission of all acutely or chronically unwell children who may have a different vitamin D status. It should be clearly stated that these findings are limited to quite a specific group.

**We have added information to better describe our sample: A convenience sample was used. For balanced representation across infant and child ages and HIV status groups, 18 mother-infant pairs with infants aged 0-5.9 months and 18 pairs with infants aged 6-11.9 months attending routine medical visits were enrolled. (lines 108-110).**

**We also added the sample as a limitation in the discussion: Additionally, we used a convenience sample that may not be representative of the population the sample size was modest and future larger studies are needed to confirm these data. (line 299)**

Line 113: The amended sentence is not grammatically correct. You could preface this by saying that infants of unconfirmed HIV status were categorized as negative ‘given the rate of perinatal transmission etc.’ Sensitivity analyses are described in the response to the reviewer comment. IT might be useful to mention that sensitivity analyses were performed which did not change the observed associations.
With all the stratification the numbers become very small and once must be careful not to overstate the findings. Caution is required in interpreting these data.

**Thank you for this comment. We have altered the sentence and added detail about the additional analysis: Given the rate of perinatal transmission of HIV in Botswana was less than 3% at the time of data collection(16), the younger infants (aged 0-5.9 months) were categorized as HIV-. Removing the younger infants did not change the results, therefore, we included them in the dataset.**

Line 118: adding “unrelated” children would make the groups explicit.

**We have added this.**

Line 125: HIV status – this is usually positive, negative or indeterminate; could this refer to the clinical and/or immunological stage of women living with HIV?

**We are also classifying how well-controlled/compliant with medication participants are (using CD4 count, viral load).**

Line 145: ‘Immunological outcomes’, like ‘an immune panel’ in abstract and ‘immunological markers’ in line is a bit misleading if you mean CD4 and VL only. The terms imply additional assays (cytokines? T cell phenotype?) For example, “Immune status was determined in all women and those children living with HIV…” may be more suitable here.

**We have changed the sentence to your recommended language. (line 147)**

Line 160: were all data normally distributed?

**Variables were assessed for normality using a Skewness and Kurtosis test (Stata) and all primary outcome variables were normally distributed. Some variables were slightly skewed according to the SK test, but quantile plots appeared normal, so variables were not transformed.**

Line 167: Why did you choose ANCOVA over regression analysis?
Did you check the model?

**We used ANCOVA primarily because we were interested in generating the adjusted means (LS means) for the variables.  In the case of the nutritional status variables, such as serum vitamin D, we were interested in the difference between groups (mothers with HIV+ vs. HIV- infants or HIV+ vs. HIV- infants and children) adjusted for the seasonal effect which is known to occur.  In the case of the growth variables in the infants and children, we were interested in what the mean differences were in the Z scores between HIV+ and HIV- groups after adjusting for the effect of age. Regression models give much the same results in terms of the significance of the groups differences in these variables, however they do not generate the adjusted means.**

Please add a description of relevant PMTCT and ART guidelines at the time as these speak to additional exposures.

**We have added the following language under methods: Between 2006-2009, more than 95% of HIV+ pregnant women in Botswana received the national standard antiretrovirals (ARVs) for prevention of mother to child transmission (PMTCT). This was all HIV infected pregnant women were eligible for triple prophylaxis/ART. HIV infected women were evaluated for ART eligibility based on laboratory testing (CD4 cell count) or clinical presentation (WHO clinical stage). Women with CD4 cell count less than 350 or WHO clinical stage 3 or 4 were started on ART through the national ARV Program as soon as possible, regardless of the stage of pregnancy. Women attending healthcare facilities that provided triple ARV prophylaxis, who were not eligible for ART were provided with triple ARV prophylaxis starting from 14 weeks of gestation. Women who chose to breastfeed continued triple ARV prophylaxis until their infants were at least six months of age and completely after weaning. Both women on ART and those that were on ARV prophylaxis were given supplementary AZT 300 mg every 3 hours during labor and delivery.**

**Similarly by 2009, >95% of infants born to HIV infected mothers were given ARV prophylaxis at birth according to national guidelines; they were given Nevirapine as a single dose as soon as possible after birth (within 72 hours) plus four weeks of AZT.**

Line 182: This is a low proportion – is ART not indicated for all pregnant women in Botswana?

**The paragraphs above explain this – this study was done at the time that ART rollout for all HIV infected pregnant women was being rolled out across Botswana.**

Line 187: As presented the sentence states that the HIV infected infants were examined in on season and the HEU in another. Is this correct?

**This is correct.**

Line 197: breastfeeding. Does this refer to current BF or ever BF?

**This refers to “ever” breast fed. We have added “ever” to this sentence.**

Line 283: The association was weak; a weak but significant positive correlation.

Reviewer 2 had also noted that the original statement is very emphatic.
Are CD4 values presented for the mothers or the infants/children? Conventionally, CD4 is presented as an absolute number in adults (and children >5years) and only as % in the under-fives.

**We understand the editor and reviewer’s concerns. We have amended the sentence to read: This correlation suggests adequate vitamin D status may be associated with improved HIV disease status.**

**Additionally, we are only referring to the infected mothers for this correlation: We found a significant, positive correlation between serum 25(OH)D and CD4% in the HIV infected mothers.**
