# Supplementary material for: Vitamin D status, nutrition and growth in HIV-infected mothers and HIV-exposed infants and children in Botswana
Source: PLoS One. 2020 Aug 13;15(8):e0236510. doi: 10.1371/journal.pone.0236510 (PMC7425960; doi:10.1371/journal.pone.0236510)
Supplement: Attachment — Submitted filename: Response to editor 062920.docx [file pone.0236510.s005.docx]

I apologize for going backwards and forwards. It is now clear that the women-infant pairs were recruited several years ago (2012 - 2013) and the samples banked. Was this part of an earlier study, perhaps with different objectives? If so, please describe the enrollment procedures of the original study and how the current sample reflects the participant profile of that study. Were the unrelated older children enrolled over the same period in 2012 - 2013? If not, please state this and justify their inclusion. From where were they recruited? As acknowledged by the authors, the sample sizes are small and it is important that there was some system supporting participant inclusion if these results are to be useful. The integrity of the comparator groups needs to be sound and the methodology explicit.

**Thank you for allowing us to clarify these details. The women-infant pairs and the unrelated children were all specifically recruited for this study which was conducted in 2012-2013. We have also amended the following sentence in the discussion to better characterize the limitations of the study: “we used a convenience sample recruited in 2012-2013 that may not be representative of the population, the sample size was modest and future larger studies are needed to confirm these data.” Although data was collected during this time period, this is the only data that describes vitamin D status and growth in Batswana infants and young children. Hence these data remain important and we hope that the publication of this study will stimulate further work in this area.**

I am still uncertain with respect to the PMTCT guidelines at the time of the study: were these the same as the 2009 guidelines referenced?

**Thank you for the opportunity to clarify. The PMTCT guidelines section has been revised to better describe the care guidelines at the time of data collection, which does reflect that ART was available to pregnant women, but implementation was not yet universal.**

Please include in your abstract and discussion that these findings reflect Vitamin D status in a population prior to the introduction of universal ART in pregnancy.

**We have included this (lines 47-48 & 300-301).**
